# Study on the Mechanism of Sarsasapogenin in Treating Precocious Puberty by Regulating the HPG Axis

**DOI:** 10.1155/2020/1978043

**Published:** 2020-08-05

**Authors:** Kaili Hu, Wenyan Sun, Yu Li, Bo Zhang, Meng Zhang, Chunyan Guo, HongSheng Chang, Xiaoling Wang

**Affiliations:** ^1^School of Chinese Materia Medica, Beijing University of Chinese Medicine, Beijing 100102, China; ^2^Beijing Children's Hospital, Capital Medical University, National Center for Children Health, Beijing 100045, China

## Abstract

The present study aims to investigate the effects and mechanisms of sarsasapogenin resistance to precocious puberty. Female Sprague Dawley rats were divided into a normal (N) group, model (M) group, leuprolide (L) group, and sarsasapogenin (Sar) group. Rats at 5 days of age were given a single subcutaneous injection of 300 micrograms of danazol to establish the precocious puberty model. After 10 days of modeling, drug intervention was started. The development of the uterus and ovary was observed by hematoxylin and eosin (HE) staining. The levels of the serum luteinizing hormone (LH), follicle-stimulating hormone (FSH), and estradiol (E2) were determined by radioimmunoassay. Also, the expressions of the hypothalamic gonadotropin releasing hormone (GnRH), Kiss-1, G protein-coupled receptor 54 (GPR54), and pituitary gonadotropin releasing hormone receptor (GnRH-R) were detected by RT-PCR. The results showed that compared with the model group, sarsasapogenin could significantly delay the opening time of vaginal, decreased uterine and ovarian coefficients, and reduced uterine wall thickness. Moreover, it can significantly downregulate the levels of serum hormones and reduce the expression of GnRH, GnRH-R, and kiss-1. In summary, our results indicate that sarsasapogenin can regulate the HPG axis through the kiss-1/GPR54 system for therapeutic precocious puberty.

## 1. Introduction

Precocious puberty, a growth and development disorder, is caused by the premature activation of GnRH neurons [1]. It is defined as the appearance of secondary sex characteristics before 8 years of age in girls and before 9 years of age in boys [2]. A Danish study showed that the prevalence of precocious puberty in girls was 0.2%, that of boys was less than 0.05%, and the prevalence of girls was much higher than boys [3]. Children with precocious puberty tend to be short in stature due to early onset of puberty, early growth and development, and shortened bone growth years [4]. In addition, precocious puberty can cause a series of psychological and physical problems in patients and may be also associated with metabolic diseases such as diabetes, cardiovascular disease, breast cancer, and prostate cancer [5, 6]. In the recent years, studies have found that the age of the onset of precocious puberty has been decreasing [7, 8], and it has become a social focus.

At present, gonadotropin releasing hormone analogue (GnRHa) is widely used to treat precocious puberty [9]. Leuprolide is the most commonly used. It acts on the pituitary in a continuous nonpulsed form, downregulates its GnRH receptor or reduces its sensitivity to the GnRH receptor, and inhibits the pituitary to secrete luteinizing hormone (LH) and follicle stimulating hormone (FSH), thus inhibiting the development of secondary sex characteristics [10, 11], achieving the effect of therapeutic precocious puberty. Although leuprolide plays a significant role in the treatment of precocious puberty, it has been found that it is easy to cause vaginal bleeding when used for the first time, with an incidence of 16%–60% [12]. Also, it can also increase the incidence of polycystic ovary syndrome [13]. In the recent years, traditional Chinese medicine has been successfully used to treat precocious puberty [14], which can achieve the purpose of delaying bone age maturity and benefit the final height of patients [15]. However, the current research on the therapeutic precocious puberty of traditional Chinese medicine is mainly focused on the compound, while for single chinses herb, especially, the effective ingredients are few.

The traditional Chinese medicine Zhimu (*Anemarrhena asphodeloides Bge.*) is a dried rhizome of Liliaceae, which has the functions of clearing heat and purging fire, nourishing yin, and moistening. Pharmacological studies show that Zhimu also has antithrombosis, ameliorating Alzheimer's disease, antitumor, anti-inflammatory, antidepression, and other effects [16, 17]. Through analyzing the relevant literature on the treatment of precocious puberty of Chinese medicine in the past 40 years, it is found that, among the precocious puberty of Chinese medicine, Zhimu is used most frequently [18]. Recent studies have found that Zhimu can directly affect GnRH neurons and inhibit GnRH mRNA expression [19]. But, the main components are unknown. Sarsasapogenin is a main active component isolated from Zhimu [20]. It has been proven that sarsasapogenin is similar in structure to the hormone backbone, which can affect reproductive function in rats and has antifertility effects [21, 22]. It has been reported that antifertility drugs may be associated with the treatment of precocious puberty [23, 24]. For example, the traditional Chinese medicine Zicao (*Radix Lithospermi*), based on its antifertility effect, has also been found to reduce serum FSH, LH, and estradiol (E2) in rats, delay the opening time of the vagina, and play a role in therapeutic precocity by inhibiting the HPG axis. Therefore, we speculate that sarsasapogenin may be one of the ingredients in the antiprecocious effect of Zhimu. To test this hypothesis, we established a danazol-induced precocious puberty model to determine the effect and mechanism of sarsasapogenin.

According to Morishita et al. [25], subcutaneous injection of danazol can induce a precocious puberty model in rats. Danazol has mild androgen and progestogen activity. Simply giving female rats progestogen or physiological doses of androgen does not advance the sexual development of female rats. The mechanism of danazol is still unclear. It had been reported that women with danazol administration did not influence serum levels of estradiol, but resulted in an increase in LH pulse amplitude in women with endometriosis [26].

## 2. Materials and Methods

### 2.1. Animals

Twenty-four SPF grade Female Sprague Dawley rats at 3 days of age in company with the maters were purchased from Beijing SiPeiFu biotechnology co., LTD. (license: SYXK(JING)2016-0038). All animals were raised in a room with 24°C constant temperature (humidity 42%) and a 12-hour light/dark cycle, with food and water available ad libitum. All experimental procedures involving the animals were approved by the Experimental Animal Ethics Committee of the Academic Committee of Beijing University of Chinese Medicine.

### 2.2. Experimental Design

The rats were randomly divided into a normal (N) group, model (M) group, leuprolid (L) group, and sarsasapogenin (Sar) group. At the 5th day of age, except the N group, the other three groups were given a single subcutaneous injection of 300 *μ*g of danazol (Yuanye Bio-Technology Co., Ltd., Shanghai, China) dissolved in 25 *μ*l vehicle of ethylene glycol-ethanol(1 : 1, v/v) [25]. After 10 days of modeling, the L group was subcutaneously injected with leuprolide (Livzon Pharmaceutical Co., Ltd., Shanghai, China) at a dose of 100 *μ*g/kg, and the Sar group was gavaged with a dose of 4 mg/kg (Yuanye Bio-Technology Co., Ltd., Shanghai, China). The N and M groups were given the same amount of saline. At the age of 20 days, the rats were inspected daily for vaginal opening; thereafter, vaginal smears were examined daily. After a regular estrous cycle, the rats in the model group were sacrificed during the diestrus stage, and other groups were also randomly sacrificed at the same time at 1 : 1. All animals were weaned on day 23.

### 2.3. Estrous Cycle Determination

Animals with vaginal openings were given daily vaginal cell smears at 8:30 am, to make sure whether the rats were entering a regular estrous cycle. Vaginal cells were collected via saline lavage and, then, stained with 4% methylene-blue (Yuanye Bio-Technology Co., Ltd., Shanghai, China). Generally, a regular estrous cycle includes proestrus, estrus, metestrus, and diestrus. Nucleated epithelial cells were predominant in the proestrus stage; cornified squamous epithelial cells were predominant in the estrus stage; both cornified squamous epithelial cells and leukocytes indicated the metestrus stage; and predominant leukocytes indicated the diestrus stage.

### 2.4. Hormonal Analysis of Serum

Rats were weighed and anesthetized by intraperitoneal injection of 10% chloral hydrate (0.3 mL/100 g) before being sacrificed. Blood samples were taken from the abdominal aorta, and serum was centrifugally collected at 4°C and stored at −20°C until analysis. Serum hormones LH, FSH, and estradiol (E_2_) levels were determined using radioimmunoassay kits (sinouk institute of biological technology Beijing, China) according to the manufacturer's specifications. The sensitivity of the E_2_ kit was 5 pg/mL, and the intra- and interassay coefficients of variation were <10% and <15.2%. The sensitivity of the LH kit was 0.2–5.0 mIU/mL, and the intra- and interassay coefficients of variation were 2.0–2.4% and 4.2–7.5%. The sensitivity of the FSH kit was 0.25 mIU/mL, and the intra- and interassay coefficients of variation were 2.2–2.5% and 3.7–8.7%.

### 2.5. Pathological Examination of the Uterus and Ovary

After the blood samples were taken, uterus and ovary samples were, then, rapidly removed from the animals and weighed, and the uterine and ovarian coefficients were calculated (mg/100 g). Subsequently, uterus and ovary samples were fixed with 10% formalin, embedded in paraffin, sectioned, and stained with hematoxylin and eosin (HE) according to the standard histological procedures. To measure the thickness of the uterine wall, we took pictures of 40x field of view of each section. We made sure each photo has the same background light. Image-pro Plus 6.0 (Media Cybernetics, Inc., Rockville, MD, USA) software was used to measure the thickness of the uterine wall (mm) by taking the 40x ruler at the lower right corner as the standard and selecting 5 points for each section. Also, the corpus luteum of each ovary section was counted under the microscope (Nikon Eclipse Ti-SR, Japan).

### 2.6. Real-Time Fluorescence Quantitative Polymerase Chain Reaction (PT-PCR) Analysis

The hypothalamus and pituitary were dissected from the brain to detect the mRNA expression of GnRH, Kiss-1, G protein-coupled receptor 54 (GPR54) in the hypothalamus and GnRH-R in the pituitary. The total RNA was extracted according to the instructions of the HiPure Total RNA Mini Kit (MAGEN), and the RNA concentration was measured by using a UV spectrophotometer (UV-2000, Unico, Shanghai, China). According to the instructions, reverse transcription was performed on a T100 Thermal Cycler PCR machine (Bio-Rad, USA) using the Reveraid First Strand cDNA Synthesis kit and the SYBR PCR master mix. Amplification and quantification were performed on a Real-Time PCR machine (Bio-Rad, USA). The RT-RCR method was as follows: initial denaturation at 95°C for 10 min, then denaturation at 95°C for 10 s, annealing at 55°C (annealing temperature of Kiss-1/GPR54 is 60°C), and extension for 30 s with a total of 50 amplification cycles. The *β*-actin gene was used as the internal standard. Using the 2^−ΔΔ*Ct*^ method to calculate the relative expression levels, the primers were synthesized by Beijing Bomad Gene Technology Co., Ltd. (Beijing, China). The primers' sequence is shown in Table 1.

### 2.7. Statistical Analysis

Data are presented as mean ± SEM, and the normal distribution and homogeneity of variance were analyzed using Student's *t*-test. The Wilcoxon rank sum test was used for data with nonnormal distribution. Statistical analysis was performed using SAS 8.2 (IBM, Armonk, NY, USA). *P* < 0.05 was considered statistically significant.

## 3. Results

### 3.1. Effect of Sarsasapogenin on the Body Weight Gain of Rats

**Figure 1:**
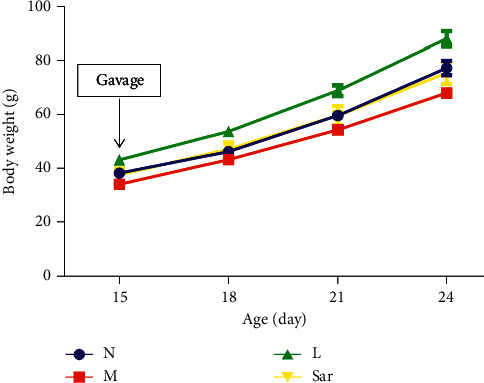
Effect of sarsasapogenin on the body weight gain of rats. Data are expressed as mean ± SEM (*n* = 6/group). N: normal group, M: model group, L: leuprolide group, and Sar: sarsasapogenin group.

The changes of body weight were observed at 15, 18, 21, and 24 days of age. The weight of 15-day-old rats was measured before administration. The result shows that there was no significant difference in these days' body weight gain between the groups (*P* > 0.05). The difference in body weight between the groups may be related to the motherhood of the female rats in each group because each group was breast-fed by its own mother (Figure 1).

### 3.2. Time of Vaginal Opening and Vaginal Cell Smear

**Figure 2:**
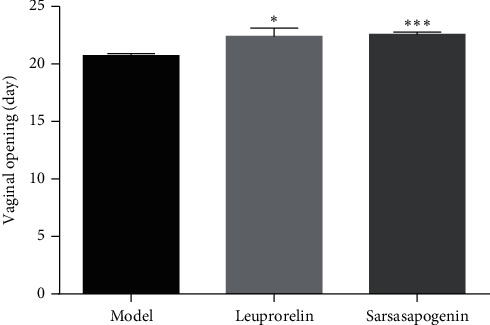
Time of vaginal opening. Data are expressed as mean ± SEM (*n* = 6/group). ^*∗*^*P* < 0.05, ^*∗∗∗*^*P* < 0.001 vs. the M group.

**Figure 3:**
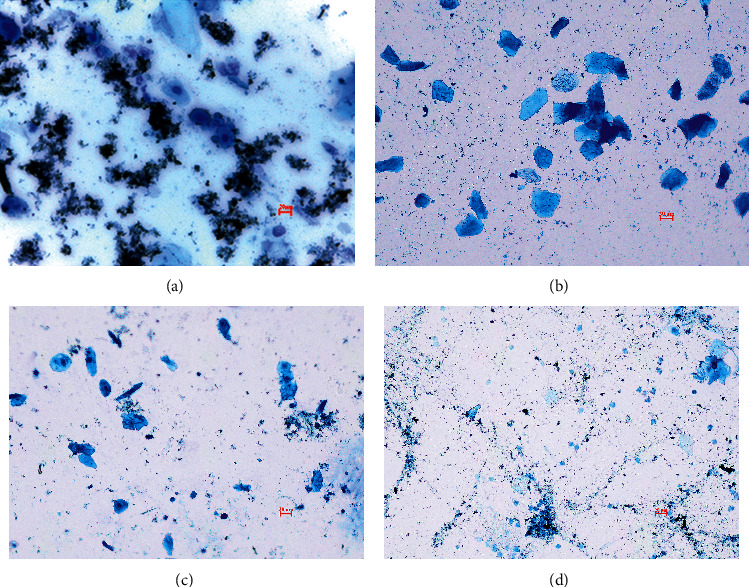
A regular estrous cycle. (a) Proestrus; (b) estrous; (c) metestrus; and (d) diestrus (20x scale = 20 *μ*m).

A vaginal opening did not appear in the N group until 30 days of age. The vaginal opening time in the M group was significantly advanced than that in N, indicating that the model of danazol-induced precocity was successfully established. Compared with the M group, the effect of delaying vaginal opening was significant in the L and Sar groups (*P* < 0.05, *P* < 0.001, respectively) (Figure 2). Also, a regular estrous cycle was as shown in Figure 3.

### 3.3. Effects of Sarsasapogenin on Uterine and Ovarian Coefficients

**Figure 4:**
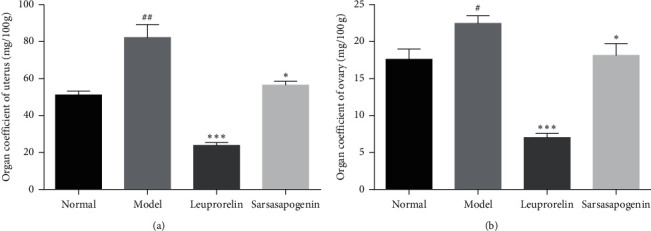
Uterine and ovarian coefficients. Data are expressed as mean ± SEM (*n* = 6/group). ^*∗*^*P* < 0.05, ^*∗∗∗*^*P* < 0.001 vs. the M group; ^#^*P* < 0.05, ^##^*P* < 0.01 vs. the N group.

The results showed that the coefficients of uteri and ovaries in the M group increased significantly than that in N group (*P* < 0.01, *P* < 0.05, respectively). Compared with the M group, the uteri and ovaries coefficients of the L and the Sar group were significantly reduced (*P* < 0.001, *P* < 0.05, respectively). It means that the L and Sar groups can inhibit the development of gonads and played a therapeutic role (Figure 4).

### 3.4. Effects of Sarsasapogenin on the Uterine Wall Thickness and Ovarian Luteinization Rate

**Figure 5:**
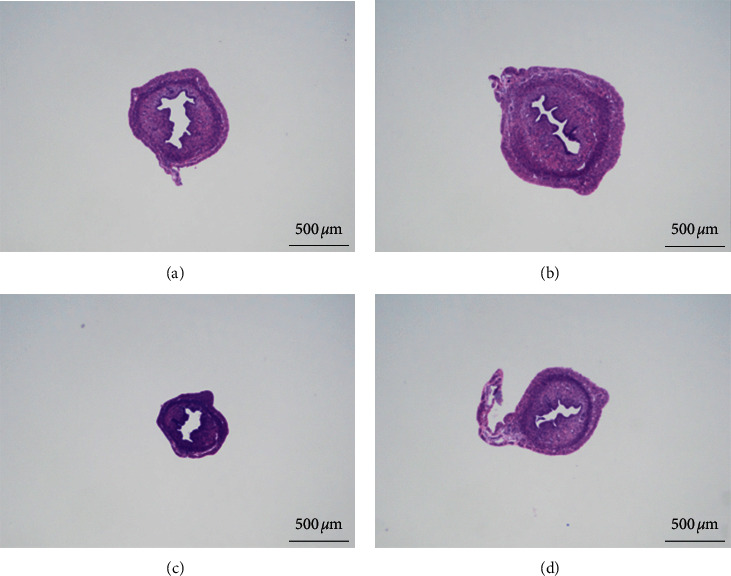
Uterine pathology section. (a) Normal group; (b) model group; (c) leuprolide group; and (d) sarsasapogenin group (40x scale = 100 *μ*m).

**Figure 6:**
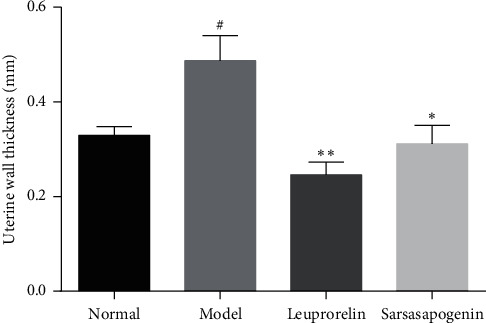
Uterine wall thickness. Data are expressed as mean ± SEM (*n* = 6/group). ^*∗*^*P* < 0.05, ^*∗∗*^*P* < 0.01 vs. the M group; ^#^*P* < 0.05 vs. the N group.

The pathological sections of the uterus showed that the M group had a clearer tissue hierarchy than the N group, and the cell morphology was normal without significant pathological changes (Figure 5). Also, the thickness of the uterine wall in the M group was significantly increased than that in N group (*P* < 0.05). Also, the uterine wall thickness in the Sar group was significantly reduced compared with that in the M group (*P* < 0.05). The effect of reducing the uterine wall thickness in the L group was the most significant (*P* < 0.01) (Figure 6). Under the microscope, the luteinizing number of ovary in each section was observed. There was only one luteinizing number in the N group, five in the M group, two in the L group, and two in the Sar group. The difference was not statistically significant; therefore, graph analysis is not performed in this study.

### 3.5. Effects of Sarsasapogenin on Serum Hormones

**Figure 7:**
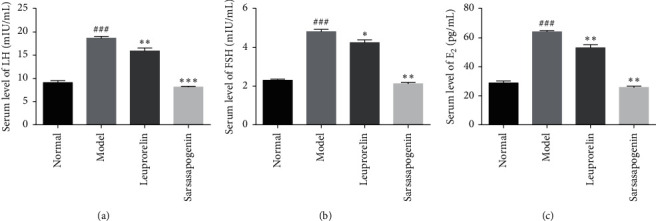
Serum hormone content. Data are expressed as mean ± SEM (*n* = 6/group). ^*∗*^*P* < 0.05, ^*∗∗*^*P* < 0.01, ^*∗∗∗*^*P* < 0.001 vs. the M group; ^###^*P* < 0.001 vs. the N group.

Compared with the N group, serum hormones in the M group were significantly increased (*P* < 0.001, respectively), consistent with the symptoms of precocious puberty. Compared with the M group, the levels of LH, FSH, and E_2_ in Sar were significantly decreased (*P* < 0.001, *P* < 0.01 and *P* < 0.01, respectively). Also, the levels of LH, FSH, and E_2_ were also significantly decreased in the L than that in the M group (*P* < 0.01, *P* < 0.05 and *P* < 0.01, respectively). In addition, from the average value of the ordinate, the inhibitory effect of sarsasapogenin on serum hormone is better than that of leuprolide (Figure 7).

### 3.6. Effects of Sarsasapogenin on Hypothalamic GnRH and Pituitary GnRH-R mRNA Expression

**Figure 8:**
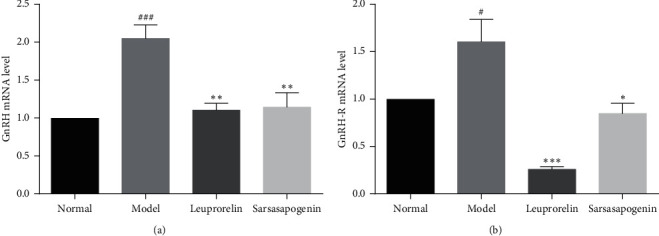
The expression levels of GnRH and GnRH-R mRNA. Data are expressed as mean ± SEM (*n* = 6/group). ^*∗*^*P* < 0.05, ^*∗∗*^*P* < 0.01, ^*∗∗∗*^*P* < 0.001 vs. the M group; ^#^*P* < 0.05, ^###^*P* < 0.001 vs. the N group.

Compared with the N group, the expression of GnRH and GnRH-R mRNA were significantly increased in the M group (*P* < 0.001, *P* < 0.05, respectively). Compared with the M group, the L group significantly reduced the expression of GnRH (*P* < 0.01). Also, it has a significant inhibitory effect on GnRH-R (*P* < 0.001), which is consistent with the mechanism of leuprolide. After treatment with sarsasapogenin, the mRNA expression of GnRH and GnRH-R decreased significantly (*P* < 0.01, *P* < 0.05, respectively). These results indicate that sarsasapogenin may act on the HPG axis to treat precocious puberty (Figure 8).

### 3.7. Effects of Sarsasapogenin on the Expression of Kiss-1 and GPR54 mRNA in the Hypothalamus

**Figure 9:**
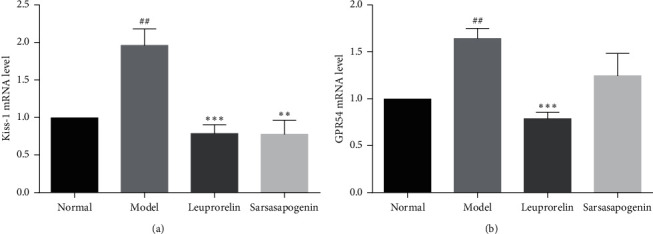
The mRNA expression of Kiss-1and GPR54 in the hypothalamus. Data are expressed as mean ± SEM (*n* = 6/group). ^*∗∗∗*^*P* < 0.001, ^*∗∗*^*P* < 0.01 vs. the M group; ^##^*P* < 0.01 vs. the N group.

Compared with the N group, the mRNA expressions of Kiss-1 and GPR54 in the M group were significantly increased (*P* < 0.01). Compared with the M group, the levels of Kiss-1 mRNA in the L and Sar groups were significantly reduced (*P* < 0.001, *P* < 0.01, respectively). However, the expression of GPR54 mRNA showed that only the L group was significantly reduced (*P* < 0.001), while there was no significant difference between Sar and M groups (Figure 9).

## 4. Discussion

The incidence of precocity has been on the rise in the recent years [27], which can be clinically subdivided into true (central) precocity and peripheral precocity, with true precocity being the most common [28]. Although the main causes of different genders are different, the development process of true precocious puberty is consistent with normal puberty [29–31]. Initiation of puberty occurs by activating the HPG axis. GnRH pulsed release promotes gonadotropin secretion. It, then, acts on the gonads and promotes the secretion of sex hormones, finally leading to the maturation of the sexual organs and entering puberty [32]. Precocious puberty is caused by the early initiation of puberty.

Puberty is a complex developmental process regulated by multiple genetic and neuroendocrine factors [33, 34]. The hypothalamus of rats is generally considered immature within 1–10 days after birth [35, 36], and the maturation of the HPG axis can be regulated by administration. Danazol, a derivative of 17*α*-acetylene testosterone, has an effect on the reproductive system of rats [25]. Studies have shown that the administration of danazol to newborn rats can rapidly promote the activation of the HPG axis [37], making them enter puberty earlier. Therefore, in this study, female SD rats at 5 days of danazol administration were used as the model to analyze the true precocious puberty.

To determine whether the model is entering puberty, we measured vaginal openings, serum hormones, and uterine and ovarian coefficients. The vaginal opening is a sign of the beginning of puberty. Our results showed that the vaginal opening of the M group was significantly advanced than that in the N group, uterine and ovarian coefficients were significantly increased, and no obvious pathological changes were found in pathological sections. This indicates that the model of precocious puberty was successfully constructed. Also, the Sar group had a significant delaying effect compared with the M group, indicating that sarsasapogenin has a therapeutic effect on precocious puberty.

As the body weight *per se* and the external environment also affect the vaginal opening, in this experiment, the external environment is consistent, and the most important influencing factor is body weight. We found no significant difference in body weight gain between the groups which suggested that sarsasapogenin had no affect on body weight. In addition, according to the critical body fat theory, a slight decrease or increase in body weight has little effect on puberty development.

LH and FSH are gonadotropins synthesized by the anterior pituitary, which have significant effects on adolescent development and gonadal and reproductive functions. Premature secretion of LH and FSH can lead to early gonadal activation [38, 39]. Estradiol is a steroid hormone secreted by the ovary [40], and its receptors are distributed in the uterus, breast, and other parts, which can promote the maturation of the sexual organs [41]. In addition, estradiol is also an important hormone regulating the growth of height during puberty, which can stimulate growth by stimulating the secretion of the growth hormone (GH) [42]. Premature estrogen secretion can accelerate bone maturation, shorten growth cycle, and reduce the final height of adults [43]. Serum hormone is the most intuitive indicator of clinical efficacy [44–46]. Our results showed that sarsasapogenin could significantly reduce serum hormone levels, and from the numerical point of view, the inhibitory effect of sarsasapogenin on serum hormone is better than that of leuprolide.

GnRH is a small decapeptide that serves as an important connection between the neural and endocrine systems [47]. It acts on the anterior pituitary gonadotropes, which express GnRH-R. Driving pituitary gonadotropic hormone release (GTH), including LH and FSH, gonadotropin acts on the ovaries or testes to secrete sex hormones such as estradiol or aldosterone, which promotes gonadal development and eventually enters puberty [48–51]. We detect the mRNA expression of GnRH and GnRH-R by RT-PCR. The results showed that sarsasapogenin could significantly reduce both of the expression, indicating that sarsasapogenin could inhibit related hormones by inhibiting the release of GnRH. However, in terms of GnRH-R inhibition, the effect of leuprolide was more significant. This is consistent with its mechanism, which has the function of inhibiting pituitary and gonadal development. In addition, continuous administration of leuprolide to replace the GnRH pulsed release will result in the downregulation of GnRH-R, which will further inhibit the release of gonadotropin [10, 52].

GnRH is the enabler and core substance of the HPG axis [53], and the activation of this neuron is regulated by a variety of neuropeptides. Many studies have shown that the Kiss-1/GPR54 system is closely related to the release of GnRH. The Kiss-1 was first discovered in malignant melanoma cells in 1996 [54], and the expression of Kiss-1 mRNA was significantly increased in pubery rats [55, 56]. By injecting kisspeptin-10 into a sheep's brain, a large amount of GnRH can be directly observed in the cerebrospinal fluid, accompanied by an increase in LH [57]. This effect is caused by the Kiss-1-encoded product, kisspeptin, which binds to the GPR54 [58]. GPR54 is a G protein-coupled receptor in the rhodopsin family [59]. People with GPR54 dysfunction develop hypogonadism. By using double-label in situ hybridization, it was found that 77% of the GnRH neurons coexpress GPR54 mRNA [60]. It means that the Kiss-1/GPR54 system may directly act on GnRH neurons to promote the secretion of GnRH.

Therefore, the mRNA expression of Kiss-1 and GPR54 were detected. The results showed that Kiss-1 mRNA in L and Sar groups were significantly reduced than those in the M group. Also, unlike leuprolide, GPR54 mRNA levels in the Sar group did not differ significantly compared to the M group. It has been reported that the effect of GPR54 on puberty development is not only related to the expression of GPR54 mRNA but also related to the functional characteristics of GPR54. Therefore, as long as the GPR54 receptor function is normal and has the property of high binding with the ligand, it can still cause the excitation of GnRH neurons [61]. So, it is not ruled out that sarsasapogenin may exert resistance and precocity through the Kiss-1/GPR54 system.

In summary, sarsasapogenin can downregulate the expression of GnRH and GnRH-R through the Kiss-1/GPR54 system, reduce serum hormone levels, and inhibit the development of gonads, thereby delaying the activation of the HPG axis and exerting the effect of therapeutic precocity. Also, sarsasapogenin is a major active ingredient in Zhimu, which can be taken orally [62, 63], is safe, and has a similar effect to leuprorelin. So, it has good development prospect.

## Figures and Tables

**Table 1:** Primers' sequence.

Name of the gene	Primer sequence (5′ to 3′)	Amplification fraction (bp)
GnRH	Forward: GGAGCTCTGGAACGTCTGATT Reverse: CAGCGTCAATGTCACACTCG	100

GnRH-R	Forward: CAGGACCCACGCAAACTACA Reverse: GGGAGTCCAGCAGATGACAA	117

Kiss-1	Forward: GCTGCTGCTTCTCCTCTGTGT Reverse: CTGTTGGCCTGTGGGTTCA	88

GPR54	Forward: GCGGCCACAGATGTCACTTT Reverse: AGGTGGGCAGCGGATAGAG	70

*β*-Actin	Forward: TGACAGGATGCAGAAGGAGA Reverse: TAGAGCCACCAATCCACACA	104

## Data Availability

The datasets used and analyzed during the current study are available from the corresponding author on reasonable request.
